# Dynamic volume magnetic domain wall imaging in grain oriented electrical steel at power frequencies with accumulative high-frame rate neutron dark-field imaging

**DOI:** 10.1038/s41598-018-33835-8

**Published:** 2018-10-25

**Authors:** Ralph P. Harti, Markus Strobl, Rudolf Schäfer, Nikolay Kardjilov, Anton S. Tremsin, Christian Grünzweig

**Affiliations:** 1 Paul Scherrer Institute, Laboratory for Neutron Scattering and Imaging, Villigen, Switzerland; 2 Leibniz-Institut für Festkörper- und Werkstoffforschung, Dresden, Germany; 3 Helmholtz-Zentrum Berlin, Institute Applied Materials, Berlin, Germany; 4 University of California at Berkeley, Space Sciences Laboratory, Berkeley, United States

## Abstract

The mobility of magnetic domains forms the link between the basic physical properties of a magnetic material and its global characteristics such as permeability and saturation field. Most commonly, surface domain structure are studied using magneto-optical Kerr microscopy. The limited information depth of approx. 20 nanometers, however, allows only for an indirect interpretation of the internal volume domain structures. Here we show how accumulative high-frame rate dynamic neutron dark-field imaging is able for the first time to visualize the dynamic of the volume magnetic domain structures in grain oriented electrical steel laminations at power frequencies. In particular we studied the volume domain structures with a spatial resolution of ∼100 μm and successfully quantified domain sizes, wall velocities, domain annihilation and its duration and domain wall multiplication in real time recordings at power frequencies of 10, 25 and 50 Hz with ±262.5 A/m and ±525 A/m (peak to peak) applied field.

## Introduction

Today’s global electricity market sizes to approximately 5 trillion watts. Large power transformers play an important role in this market, being indispensable for transporting electricity along high voltage transmission lines. In 2010, the United States’ the power demand for only large power transformers (maximum capacity rating greater than or equal to 100 MW) alone was 127 309 MW for as reported in^1^. As such, even a small gain in efficiency, by the sheer size of the market, immediately results in a huge energy and cost saving. E.g. for the number quoted above a 0.2% increase in efficiency already compares to an average nuclear power plant’s power production of 1000 MW (electrical power).

Grain-oriented electrical steels (GOES) which are typically used in transformer cores are so-called soft magnetic materials where bulk magnetic domain structure and the mobility of the domains walls determine the efficiency of transformers.

Domain structures are commonly characterized statically with surface sensitive techniques such as magneto-optical Kerr microscopy^2^. As transformers are operated at frequencies of power networks the analysis of their surface domains is done with dynamic observation techniques like excitation/scanning synchronization^3^ and sampling stroboscopy^4^. Information about domain wall motion, wall spacing, bowing, nucleation and domain refinement of GOES at power frequencies are reported in^5^ where stroboscopic and even real time experiments are reported all together using light-based Kerr microscopy techniques. The disadvantage of Kerr microscopy is that the insulating coating, which is used in most transformer steel cores, has to be removed for the analysis to expose the surface of the steel. That removal of coating changes the underlying bulk and surface domain structure^6^. Surface dynamic domain observation can be done by high-voltage scanning electron microscope^7,8^ with insulating coating. Recent developments in imaging with magneto-optical indicator films (MOIF) make it possible to perform dynamic investigations at a frequency of 50 Hz while keeping the coating intact^9^. However, while MOIF enables the visual analysis of domain wall motion it is still limited to surface domain behavior. The experimental accessibility of magnetic domains within the volume of steel is limited to a special “freeze-in” technique, the Libovicky method^10,11^, which does not allow obtaining dynamic volume domain information. The combination of dynamic domain investigations with volume information will be presented in this article and will be made now accessible for experimentalists.

The unique capability of neutrons to penetrate materials opaque to other non-destructive techniques, e.g. utilizing on light or electrons, makes it possible to study bulk behavior of magnetic domain walls while keeping the insulating coating intact and thus without modifying the domain structure. The dark-field image (DFI) of neutron grating interferometry (nGI)^9^ recently emerged as a valuable complementary technique to the established domain observation methods because it is the only technique available that enables the spatially resolved analysis of bulk magnetic domain walls^5,12–15^ deep in the volume of materials and thus provides unique information. The contrast is based on the small-angle scattering of neutrons at domain walls and the consequent disruption of a predefined interference pattern. The potential has been shown for static radiography^12^ and tomography^16^ as well as magnetic domain response studies in static measurements with stepwise changing magnetic field excitation^14,17^. Furthermore time averaged measurements in alternating fields were performed and have been published by Betz *et al*.^15^ for various power frequencies and fields. The typically long exposure times of several minutes for a single DFI in nGI experiments with standard scintillator based detector systems did not allow to study the dynamic properties of magnetic domain structures in bulk GOES at power frequencies.

**Figure 1:**
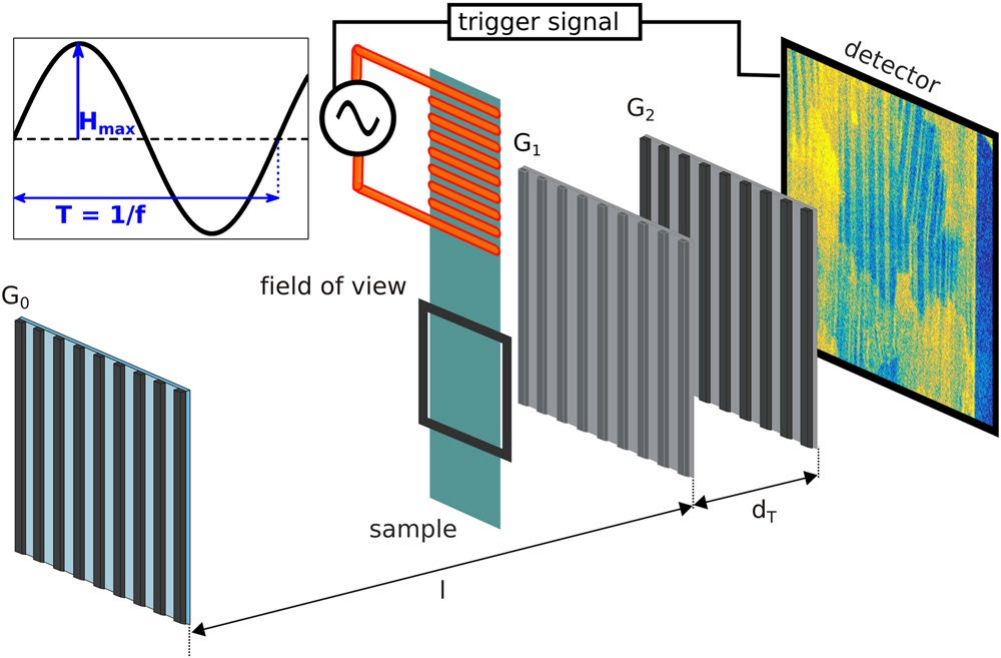
Neutron grating interferometer setup for accumulative high-frame rate dynamic neutron dark-field imaging to study dynamic volume magnetic domain wall motion. G0 and G2 are absorption gratings and G1 is a phase grating. The detector and sample are triggered with the same signal and a sinusoidal magnetic field is applied to the GOES sample.

In this article, we introduce accumulative high-frame rate dynamic neutron dark-field imaging (ADDI) where a standard nGI setup is combined with a fast neutron counting detector providing both time and position for each detected neutron^18,19^. The schematical setup is shown in Fig. 1.

The high frame rate read-out electronics allow us to measure the time of neutron detection relative to the phase of the applied magnetic field and thus synchronize the detector with sample dynamics and accumulate the time resolved signal for a large number of periods of repetitive processes. This accumulation of periods allowed us to record up to 844 time bins in a 50 Hz cycle resulting in μs time resolution. Thus events are recorded for all phases of the repetitive dynamic process without rejection of majority of events in case of traditional stroboscopic imaging. Specifically tailored data reduction was developed to quantitatively study the dynamic behavior of bulk magnetic domain walls under realistic conditions. We applied the technique to observe domains in GOES with spatial resolution of ~100 μm and successfully obtain domain size, wall velocity, domain annihilation and its duration and domain wall multiplication during magnetization in real time recordings at power frequencies of 10, 25 and 50 Hz with ±262.5 A/m and ±525 A/m (peak to peak) applied field. The GOES sample studied in this paper is in accordance with the standard grade EN 10107: M 100-30p (Thyssen Krupp powerCore H, Grade: H 100-30, Core losses at 1.7 and 50 Hz: 1.00 W = kg), and has a width of 30 mm, a length of 300 mm and a thickness of 270 μm. It is covered with an isolation layer consisting of forsterite (Mg_2_SiO_4_) and phosphate. The samples were cut into Epstein strips and annealed at 800 °C for 2 hours in nitrogen atmosphere to undergo stress-relief annealing. As the coating barely attenuates neutrons nGI is ideally suited for the study of samples with intact coating.

## Data Generation, Reduction and Analysis Routine

### Data generation

**Figure 2:**
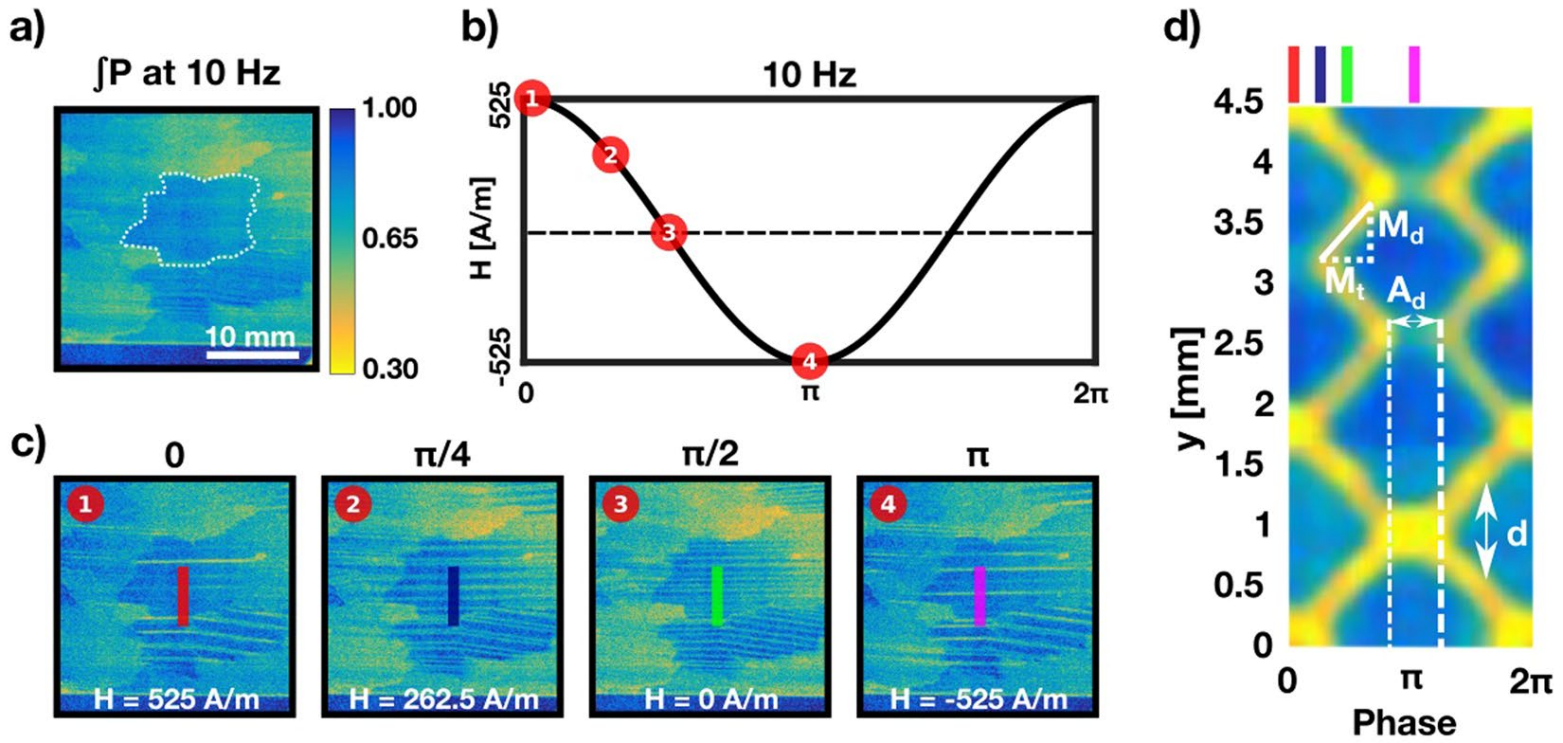
Data recording of mobile magnetic domains and extraction of quantifiable values. (**a**) Image of the sample integrated over all 62 time frames at ±525 A/m and 10 Hz. The white, dashed outline indicates the area in the sample used for further, detailed analysis. (**b**) Schematic of the applied sinusoidal field. Representatively we choose 4 characteristic images out 62 indicated by red dots. (**c**) Time resolved dark-field images corresponding to red dots. (**d**) 2D representation of the magnetic domain wall movement with the horizontal axis representing time and the vertical axis the location within the line scans. The measure parameters used for the quantitative analysis are indicated. *M*_*d*_: Measurement distance; *M*_*t*_: Measurement time; *A*_*d*_: Annihilation duration; *d*: Domain size.

Figure 2a shows a time averaged image that was generated by integration over all recorded time frames. The area marked by the white dashed outline indicates a well oriented, individual, grain that will representatively be used for domain wall motion analysis throughout the rest of the work. The absence of underlying supplementary (Lancet) domains in the grain enables the best visibility for the individual bulk domain walls under investigation. In such time averaged images (Fig. 2a) the motion of the bulk magnetic domain walls causes motion blurring and the domain walls appear as a veil, in accordance with Betz *et al*.^14,16^.

ADDI, through a combination of a fast neutron counting detector with high time and spatial resolution and a sample environment creating a specific magnetic field in the sample, allows us to extract time-resolved dark-field images with high resolution within the period of an applied alternating H-field. A few corresponding representative time slices over a period, as indicated by the red dots in Fig. 2b for 10 Hz and an applied field of ±525 A/m are presented in Fig. 2c. Image 1 at phase 0 shows the sample at H = 525 A/m and marks the start of the applied sinusoidal field. Image 2 resembles H = 262.5 A/m and represents the state of the sample at a phase of π/4. Image 3, at π/2, shows the sample state at no applied field, H = 0 A/m, and Image 4 at the low-point of H = −525 A/m indicating the process phase of π. Millisecond time resolution enables the observations of the domain walls without significant motion blurring. The observed domain walls are resolved individually and are horizontally elongated throughout the crystallographic grain and beyond. Their signal is visualized by the horizontal, yellow lines. The origin of the signal has been extensively discussed, amongst others, in Grünzweig *et al*.^20^. Already in the four exemplary images we observe the specific dynamic behavior of the magnetic domain walls. Image 1 represents a phase of the applied field at maximum effective field where some of the magnetic domain walls vanish, which indicates the annihilation of domains antiparallel to the applied field. The remaining domain walls exhibit stronger signals as a consequence of two individual domains being close together and appearing as one. The decrease in effective field at π/4 causes the domain walls to form pairs that are closer to each other, indicating growth of additional domains. No effective field at π/2 exhibits evenly spaced domains within the area marked by the dashed white line. A behavior similar to Image 1 can be observed in Image 4 at the phase of π within the period with the maximum effective field at opposite sign than at phase 0.

### Data reduction

**Figure 3:**
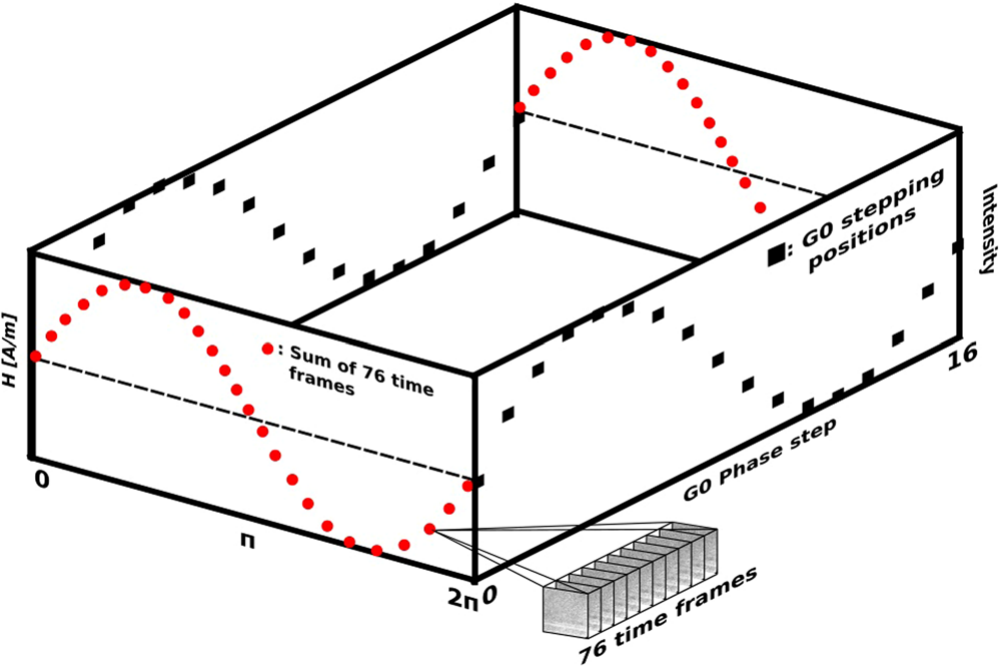
Visualization of the data recording matrix. The red points indicate time intervals composed of 76 individual time frames of the single DFIs within an applied sinusoidal field (H[A/m]). In reality the coverage was realized by a running average procedure causing the time intervals to overlap. The black squares indicate the steps within a G0 phase step. A total of 17 steps was recorded during stepping of one full period of G0. At each time interval (red points) a full G0 phase step was recorded with again each individual projection (black square) composed of 76 time frames.

The high time resolution accumulative nature of the data allows us to tune time resolution and trade it for statistics quality in post processing. It is hence possible to study the domain wall motion with some flexibility with respect to time resolution, which allows to optimize observations with regards to the significance of features in the images. Figure 3 illustrates the data recording matrix of time resolved neutron grating interferometry experiments. At every point of the applied field (H [A/m]) a full set of 17 G0 phase steps has been recorded.

The applied field was recorded with 862 individual time frames for 10 Hz, 684 for 25 Hz and 844 for 50 Hz. In order to increase the statistics in one image of the G0 phase step 76 individual time frames were summed. Each of the 17 steps of the stepped grating is thus composed of 76 time frames and the coverage of the full applied sinusoidal field was realized by a running average approach making the full data treatment a 3 step procedure: (i) Time frame summation according to phase, (ii) Running time average and (iii) Batch data reduction^18^. The summation of individual time frames (i) creates an image that is then used for the data reduction process and makes it possible to tune the statistics in the resulting DFIs. We opt for the summation of 76 images to generate one G0 phase stepping image. For the creation of the time resolved data set we use a running average approach (ii) with a step width of 14 images as a compromise between smoothness of the result and data volume. This means that for the first DFI of the time series we take the sum of time frames 0–75 as defined before. For the second DFI we sum images 13 to 88. This procedure is then continued to cover the whole set of up to 862 time frames. The summation and running average steps create relatively large sets of images that are then analyzed by a batch data treatment (iii) software based on recently developed in-house software^21^ which enables the application of common nGI data reduction algorithms to large datasets. The flexibility of that type of data treatment allows us to tune for optimal statistics and time resolution and gives us the possibility to analyze the movement of magnetic domain walls with an effective time resolution of 8.8 ms for 10 Hz, 4.4 ms for 25 Hz and 1.8 ms for 50 Hz, as well as spatial resolution with a pixel size of 50 μm.

Further measurements were conducted applying a field of ±525 A/m at frequencies of 10 Hz, 25 Hz and 50 Hz as well as ±262.5 A/m at 50 Hz. As a compromise between statistics and time resolution we opted for time intervals of 8.8 ms for 10 Hz, 11 ms for 25 Hz and 1.8 ms for 50 Hz during post processing. The videos of the behavior of the magnetic domain walls in response to the applied fields can be found in Supplementary Fig. S1.

### Data analysis routine

The variation of the applied frequency and field strength in *in-situ* time resolved measurements displaying the individual domain walls enabled us to characterize bulk domain wall movements by extracting parameters such as number of domains, domain size, domain wall velocity as well as domain wall annihilation duration.

Parameter extraction and further analysis is focused on the grain identified in Fig. 2a and indicated by the white dashed outline. The analysis of the time resolved data is based on extracting line profiles from the individual DFIs along a vertical line, perpendicular to the domain walls, from each time slice as indicated by colored vertical lines in Fig. 2c. Such a line profile was extracted for every time frame within the full period of the applied field. In Fig. 2d the intensity line profiles are plotted along the vertical axis and are combined along the horizontal axis. The horizontal axis represents the time elapsing over one period of the applied sinusoidal AC field. The differently colored lines added on top of the resulting carpet indicate the specific of the applied stimulus, according to the images in Fig. 2c. This representation of the dataset allows us to visually inspect the domain wall movement within a 2D representation and extract qualitative information straightforwardly. Image summation and running average data treatment determines the resolution in time along the x-axis of the plot and resolution was finally chosen with 62 time bins per period for 10 Hz, 49 bins for 25 Hz and 60 bins for 50 Hz. The differences in number of increments per period from one frequency to another is a consequence of a varying number of initial time frames while keeping summation and running average the same throughout the datasets.

## Quantitative Analysis of Bulk Domain Structures

2D plots, such as the one in Fig. 2d, enable us to straightforwardly deduce the number of domains *n*, domain size *d*, domain wall velocity *v* and annihilation duration *A*_*d*_. The annihilation duration describes the time period in which some of the magnetic domain walls annihilate to transport magnetic flux at relatively high fields. Annihilation can be observed in the interval indicated by the white, dashed lines in Fig. 2d, at a height of 2.75 mm, whereas no annihilation, but an increases in signal strength, can be observed at a height of 1 mm. *d* indicates the domain wall distance corresponding to the domain width and the angle α is directly correlated with the domain wall velocity *v*. *A*_*d*_ and *d* are directly measured physical quantities and *v* is represented by the slope of the solid line of the triangle in Fig. 2d. In order to extract *v* we use following equation:

1 $$v=\frac{{M}_{d}}{{M}_{t}}.$$

The movement distance *M*_*d*_ as well as the movement time *M*_*t*_ are defined in number of pixels in the plot and are indicated in Fig. 2d. While the number of pixels for *M*_*d*_ represents a length determined by the pixel size of the detector (50 µm), the number of pixels for *M*_*t*_ represents a time and is defined by the time bin duration represented by each pixel in horizontal direction. It varies between measurements with the different applied frequency as described before.

The velocity of the bulk magnetic domain walls is determined by the applied frequency and higher applied frequencies naturally cause the domain walls to move faster. To make domain wall velocities comparable throughout varying applied frequencies we introduce the frequency normalised volume domain wall velocity *v*_*n*_ defined by

2 $${v}_{n}=\frac{v}{f}$$

with *f* being the applied frequency.

**Figure 4:**
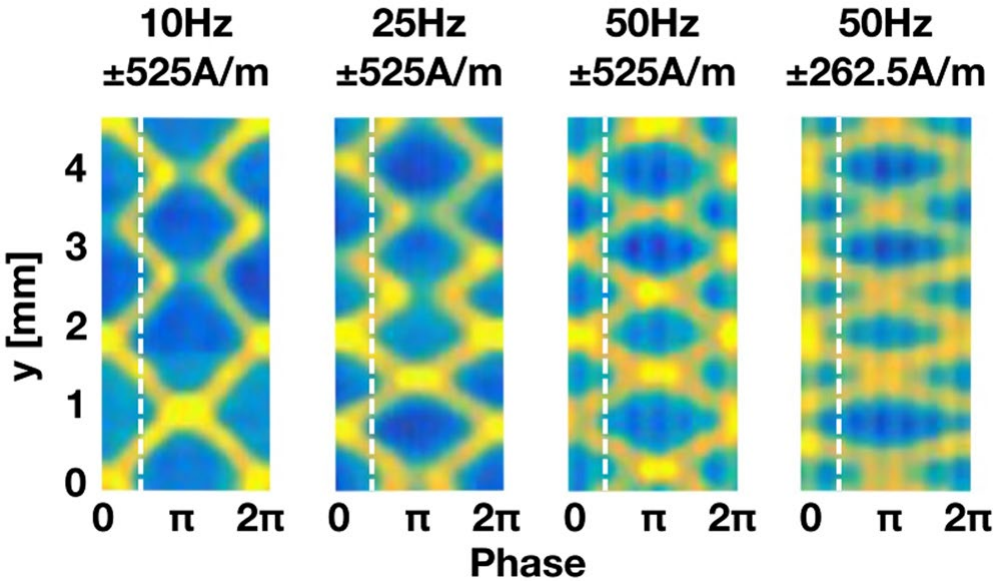
Quantitative analysis of bulk magnetic domain motion: Visual comparison of applied field/frequency combinations. The plots are created according to Fig. 2d and are the visual representation of the dynamic behaviour of magnetic domain walls in grain-oriented iron silicon high-permeability alloys. The white dashed line marks the zero-field crossing.

Visual comparison of the extracted plots for the varied experimental conditions can be done based on Fig. 4. One directly observable feature is the decrease of domain width *d* for increased frequency at constant field (plot 1–3) and decreased fields for constant frequency (plot 3–4). The white dashed line marks the first zero-field crossing within one period and the time at which d is measured. It is clearly visible that a reduction of the domain wall spacing *d* is found for increasing frequency. Another feature is the disappearance of some volume domain walls (yellow lines), indicating annihilation. Annihilations can be observed around phases of maximum effective applied field, i.e. phases of π as well as 0 and 2π, for 10 Hz and 25 Hz at ±525 A/m. At a frequency of 50 Hz no annihilation can be observed for both applied fields. For frequency independent comparison of the annihilation duration we introduce the frequency normalised annihilation duration ${A}_{d}^{n}$ in analogy to *v*_*n*_ defined by

3 $${A}_{d}^{n}={A}_{d}\cdot f.$$

**Figure 5:**
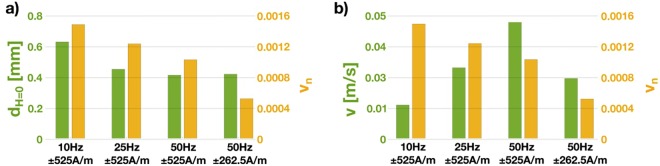
Visual comparison of physical parameters extracted from four different field/frequency combinations. The x-axis characterises the applied external field (field [A/m]/frequency [Hz]). The orange bars correspond to the right y-axis and the green bars to the left axis.

For a quantitative interpretation of the results we extracted the characteristic parameters for each frequency/field combination and summarized them in Fig. 5. The values of domain wall velocity *v*, *v*_*n*_ and domain size *d* presented in the table are an average for the domain walls observed in the plots as shown in Fig. S2. Here the individual measurements can be found including the position of the measured domain walls. From each plot, we extracted six different *d* values and averaged them to present the average domain size in Fig. 5. In case of the average domain wall velocity eight different domain walls have been measured and the average is presented.

The decreasing average domain width *d* with increasing frequency from 10 to 50 Hz (Fig. 5a) is due to domain refinement. This behavior is a consequence of the increased domain wall velocity, which causes larger damping effects^14^ that are compensated by the formation of smaller domains. As *d* is a measure for the domain width at zero effective applied field (smallest domain size throughout the process) no change of the domain size from ±525 A/m to ±262.5 A/m, at 50 Hz, can be observed. This is a consequence of the unchanged number of domain walls with decreasing the applied field. Even though the number of domain walls remains unchanged the moving distance *M*_*d*_ is reduced due to the lower excitation field and the consequentially lower magnetic flux that needs to be transported. This behavior is also reflected by the decrease in *v*_*n*_ (Fig. 5a,b) for the reduced field at 50 Hz. While the excitation frequency stays the same the travelled path is reduced, resulting in small *v*_*n*_.

A continuous increase of *v* with increased frequency (Fig. 5b) shows the ability of the domain walls to follow the applied frequency without delays, thus indicating little eddy current losses in the sample. A decrease of *v* after a reduction in applied field from ±525 A/m to ±262.5 A/m is a consequence of a smaller *M*_*d*_ at same frequency.

*A*_*d*_ for the measurements at 10 Hz and 25 Hz are 0.0282 sec and 0.00975 sec respectively with ${A}_{d}^{n}$of 0.282 and 0.26. While the absolute values show a decrease correlated to the increase in frequency, ${A}_{d}^{n}$does not change much from 10 Hz to 25 Hz. This indicates that, at both frequencies, the domain walls are able to follow the applied field without delay and thus reach the point of annihilation at the same times within the applied sinusoidal field.

## Conclusion

In conclusion, we combined the first time resolved studies of mobile volume magnetic domain walls utilizing nGI with a visual representation that allowed us to quantify the movement of domain walls in grain oriented electrical steels while keeping the coating of the applied material intact. This experimental approach gave insight into the dynamic behavior of bulk magnetic domain walls that was previously inaccessible. The type of analysis presented here has the potential to further the understanding of the inner workings of transformers as well as mobile domain wall movements in magnetic materials where the bulk domain structure is of interest. In particular such quantitative macroscopic results can serve as input parameters to develop magnetization and loss theories and simulations to further improve the efficiency of grain-oriented electrical steels, but also other magnetic materials.

## Methods

### Sample environment

The magnetization frame is a single sheet magnet with a flux closure by laminated yoke^22^ to study electrical steel sheets in Epstein geometry with sample dimension of 30 mm × 300 mm and variable sheet thicknesses. The magnetization frame was combined with a Hero PA2063A-S power supply that allowed us to vary applied frequency and field strength separately during the experiments.

### Neutron grating interferometry

The experimental work presented in this letter was conducted at the CONRAD-2 neutron imaging beamline at the Helmholtz Center in Berlin^23,24^.

The neutron grating interferometer setup, schematically shown in Fig. 1, was realized with a G0 period of 791 μm, a G1 period of 7.96 μm and a G2 period of 4 μm. The distance between G0 and G1 was 4.5 m and the distance between G1 and G2 22.7 mm, making it a first Talbot order setup for 3.5 Å. In order to fully utilize the beam intensity, the experiments were conducted with a white beam, leading to a measured visibility of 14%. The phase stepping needed for the DFI signal generation was realized by stepping G0 with 17 positions for open beam and sample images, covering one full period of G0.

### High frame-rate neutron imaging detector

A time resolved neutron imaging detector consisting of Microchannel Plates (MCPs) neutron converters and electron multiplier encoded by a Timepix readout^25^ was used to capture sample dynamics^18,19^. The MCP detector’s maximum frame rate is ~1200 frames per second, at 55 μm pixel size, with each detected neutron time tagged with resolution as good as ~1 μs. That timing resolution enables very flexible post-processing by binning the events to desired time intervals. Our experimental setup allowed ample time resolution for measurements at 10 Hz, 25 Hz and 50 Hz applied field. We recorded 862 time frames per period for 10 Hz, 684 time frames for 25 Hz and 844 time frames for 50 Hz, leading to time intervals below ms for individual frames.

## Electronic supplementary material

Supplementary Matieral

Video S3a

Video S3b

Video S3c

Video S3d
