# Supplementary material for: Dynamic volume magnetic domain wall imaging in grain oriented electrical steel at power frequencies with accumulative high-frame rate neutron dark-field imaging
Source: Sci Rep. 2018 Oct 25;8:15754. doi: 10.1038/s41598-018-33835-8 (PMC6202369; doi:10.1038/s41598-018-33835-8)
Supplement: Supplementary file 1 — Supplementary Matieral [file 41598_2018_33835_MOESM1_ESM.docx]

Supplementary Information

Dynamic volume magnetic domain wall imaging

in grain oriented electrical steel at power frequencies

with accumulative high-frame rate neutron dark-field imaging

Ralph P. Harti^1^, Markus Strobl^1^, Rudolf Schäfer^2^, Nikolay Kardjilov^3^, Anton S. Tremsin^4^, and Christian Grünzweig^1^*

1. Paul Scherrer Institute, Laboratory for Neutron Scattering and Imaging, Villigen, Switzerland
2. Leibniz-Institut für Festkörper- und Werkstoffforschung, Dresden, Germany
3. Helmholtz-Zentrum Berlin, Institute Applied Materials, Berlin, Germany
4. University of California at Berkeley, Space Sciences Laboratory, Berkeley, United States

***corresponding author: christian.gruenzweig@psi.ch**

**Supplementary Material S1: Sample**

The GOES sample studied in this communication is in accordance with the standard grade EN 10107: M 100-30p (Thyssen Krupp powerCore H, Grade: H 100-30, Core losses at 1.7 and 50 Hz: 1.00 W=kg), and has a width of 30 mm, a length of 300 mm and a thickness of 270 μm. It is covered with an isolation layer consisting of forsterite (Mg_2_SiO_4_) and phosphate. The samples were cut into Epstein strips and annealed at 800 °C for 2 hours in nitrogen atmosphere to undergo stress-relief annealing. As the coating barely attenuates neutrons nGI is ideally suited for the study of samples with intact coating.

**Figure S2.** Neutron grating interferometer setup. G0 and G2 are absorption gratings and G1 is a phase grating. The detector and sample are triggered with the same signal and a sinusoidal magnetic field is applied to the sample.


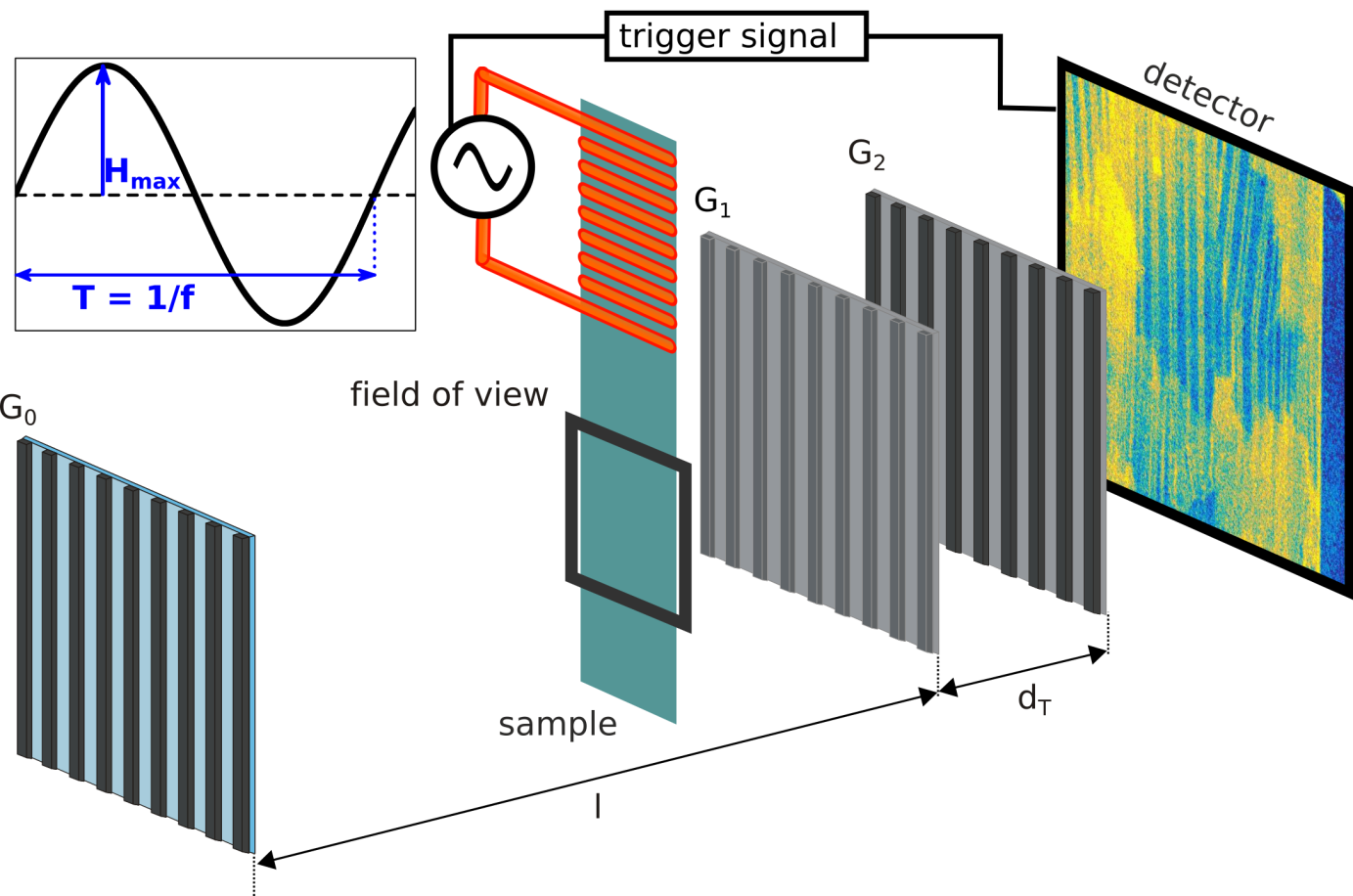


**Figure S3.** Thumbnails for the recorded videos available for download. Each of the four images represents the first frame of the recorded videos and represents the state of the sample at the highest applied field for each measurement.


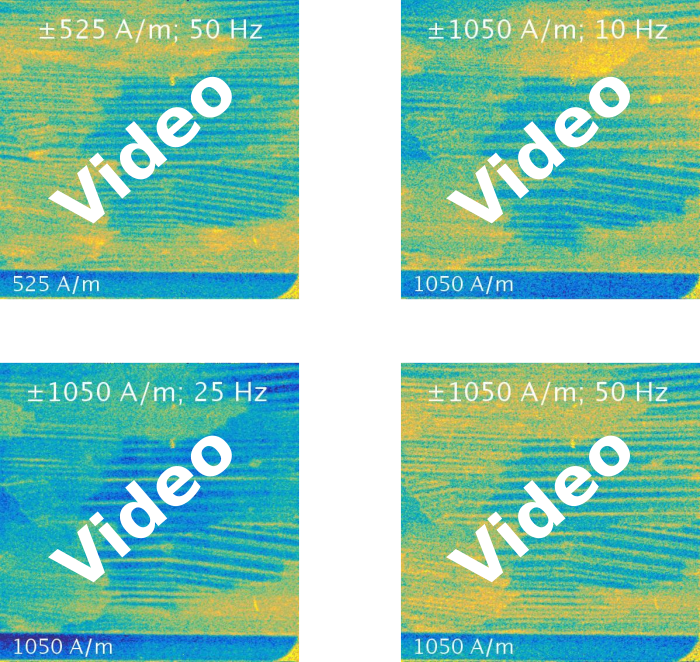


**Supplementary Material S4: Data reduction**

Figure S5 visualizes the data recording matrix of time resolved neutron grating interferometry experiments. At every point of the applied field (H [A/m]) a full set of 17 G0 phase steps has been recorded. The applied field was recorded with 862 individual time frames for 10Hz, 684 for 25 Hz and 844 for 50 Hz. In order to increase the statistics in one image of the G0 phase step 76 individual time frames were summed. Each of the 17 steps of the stepped grating is thus composed of 76 time frames and the coverage of the full applied sinusoidal field was realized by a running average approach making the full data treatment a 3 step procedure: (i) Time frame summation according to phase, (ii) Running time average and (iii) Batch data reduction. The summation of individual time frames (i) creates an image that is then used for the data reduction process and makes it possible to tune the statistics in the resulting DFIs. We opt for the summation of 76 images to generate one G0 phase stepping image. For the creation of the time resolved data set we use a running average approach (ii) with a step width of 14 images as a compromise between smoothness of the result and data volume. This means that for the first DFI of the time series we take the sum of time frames 0 - 75 as defined before. For the second DFI we sum images 13 to 88. This procedure is then continued to cover the whole set of up to 862 time frames. The summation and running average steps create relatively large sets of images that are then analyzed by a batch data treatment (iii) software based on recently developed in-house software^[[1]](#footnote-1)^ which enables the application of common nGI data reduction algorithms^[[2]](#footnote-2)^ to large datasets. The flexibility of that type of data treatment allows us to tune for optimal statistics and time resolution and gives us the possibility to analyze the movement of magnetic domain walls with an effective time resolution of 8.8 ms for 10 Hz, 4.4 ms for 25 Hz and 1.8 ms for 50 Hz, as well as spatial resolution with a pixel size of 50 μm.

**Figure S5.** Visualization of the data recording matrix. The red points indicate time intervals composed of 76 individual time frames of the single DFIs within an applied sinusoidal field (H[A/m]). In reality the coverage was realized by a running average procedure causing the time intervals to overlap. The black squares indicate the steps within a G0 phase step. A total of 17 steps was recorded during stepping of one full period of G0. At each time interval (red points) a full G0 phase step was recorded with again each individual projection (black square) composed of 76 time frames.


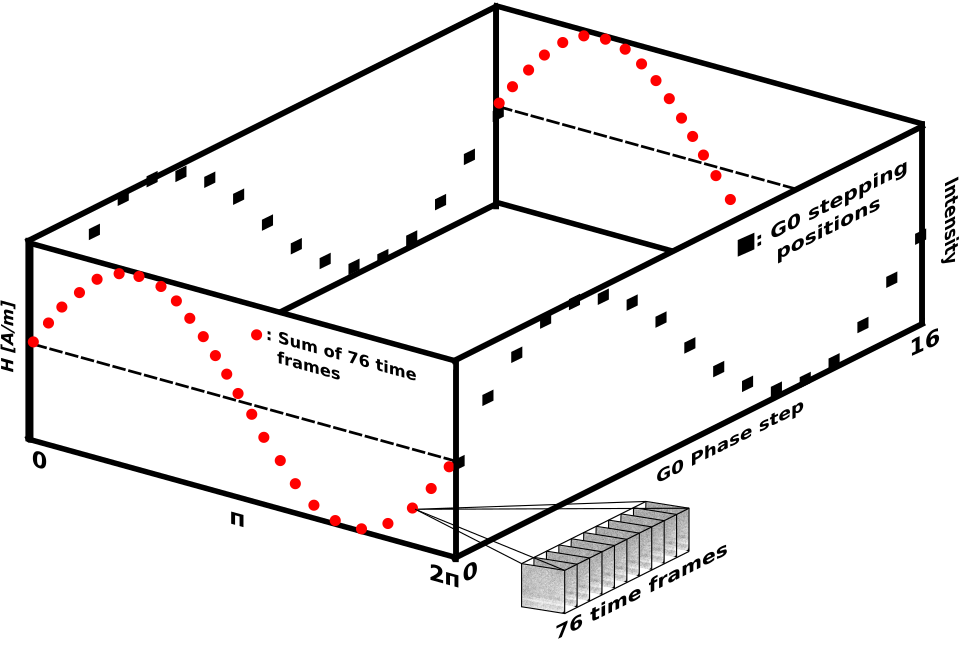


**Figure S6.** Measurement locations for the individual measurements with the respective values. Lower case letters indicate the measurement location of the domain size *d* and upper case letters indicate the domains from which the angle α was extracted. The averages of these values are presented in the table in Figure 3 in the manuscript.


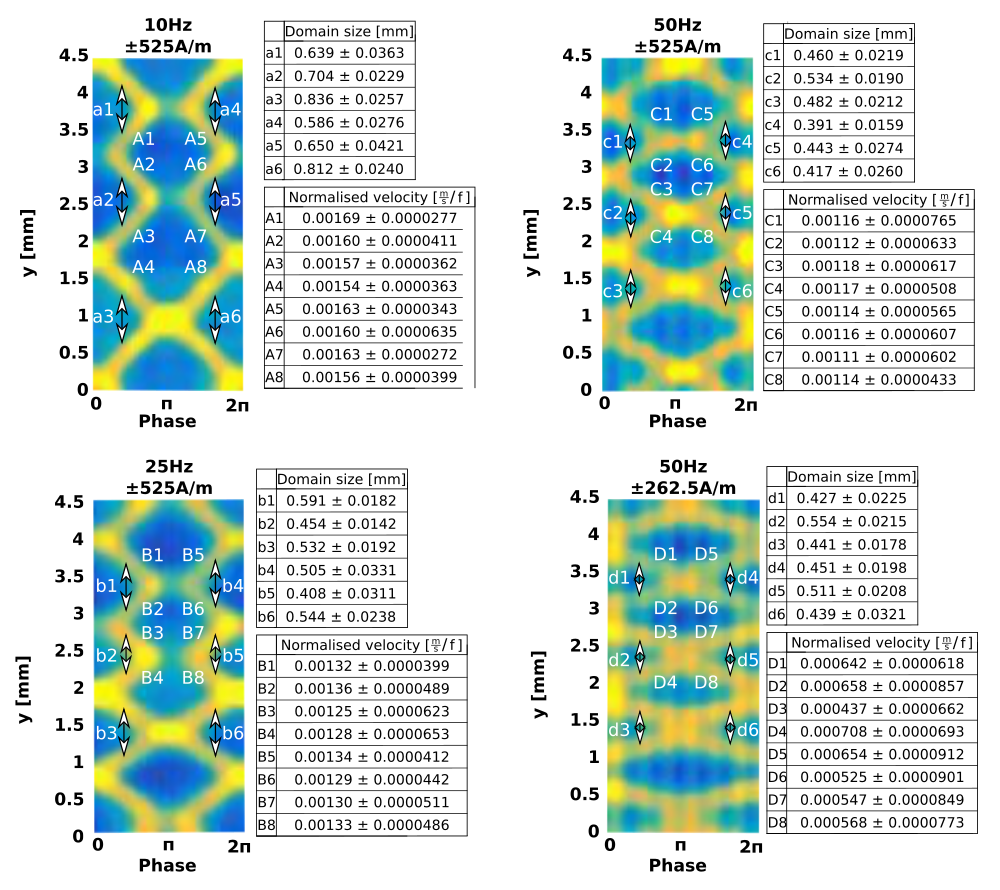


1. R. P. Harti and J. Valsecchi, Tapy 0.2 (2017), 10.5281/ZENODO.803047. [↑](#footnote-ref-1)
2. S. Marathe, L. Assoufid, X. Xiao, K. Ham, W. W. Johnson, and L. G. Butler, *The Review of scientific instruments* **85**, 013704 (2014). [↑](#footnote-ref-2)
